# Vascular Remodeling, Oxidative Stress, and Disrupted PPAR*γ* Expression in Rats of Long-Term Hyperhomocysteinemia with Metabolic Disturbance

**DOI:** 10.1155/2018/6738703

**Published:** 2018-01-15

**Authors:** Yajing Huo, Xuqing Wu, Jing Ding, Yang Geng, Weiwei Qiao, Anyan Ge, Cen Guo, Jianing Lv, Haifeng Bao, Wei Fan

**Affiliations:** ^1^Department of Neurology, Zhongshan Hospital, Fudan University, Shanghai 200032, China; ^2^Department of Laboratory Animal Science, Fudan University, Shanghai 200032, China

## Abstract

Hyperhomocysteinemia, a risk factor for vascular disease, is associated with metabolic syndrome. Our study was aimed at exploring the effect of long-term hyperhomocysteinemia with metabolic disturbances on vascular remodeling. We also studied oxidative stress and expression of PPAR*γ* in the coronary arteriole as a possible mechanism underlying vascular remodeling. Rats were treated with standard rodent chow (Control) or diet enriched in methionine (Met) for 48 weeks. Plasma homocysteine, blood glucose, serum lipids, malondialdehyde (MDA), superoxide dismutase (SOD), and nitric oxide (NO) levels were measured. Coronary arteriolar and carotid arterial remodeling was assessed by histomorphometric techniques and the expression of PPAR*γ* in vessel wall was investigated. In Met group, an increase in the level of fasting blood glucose, serum triglyceride, total cholesterol, MDA, and NO, a decline in the serum SOD level, and increased collagen deposition in coronary and carotid arteries were found. Moreover, we detected decreased expression of PPAR*γ* in the coronary arterioles in Met group. In summary, our study revealed metabolic disturbances in this model of long-term hyperhomocysteinemia together with vascular remodeling and suggested that impaired oxidative stress, endothelium dysfunction, and decreased PPAR*γ* expression in the vessel wall could be underlying mechanisms.

## 1. Introduction

Hyperhomocysteinemia is an important risk factor for atherosclerosis [1]. Several clinical studies have shown that hyperhomocysteinemia was independently associated with increased vascular disease risk [2–4]. The previous study from our group has also demonstrated that elevated homocysteine correlated with severity and prognosis in patients with atherothrombotic stroke [5]. However, the precise mechanism of hyperhomocysteinemia with atherosclerosis has not been well elucidated [6].

Several animal studies have demonstrated that hyperhomocysteinemia could induce vascular remodeling [7–9]. Some clinical studies also showed that hyperhomocysteinemia was associated with increased carotid artery wall thickness in human beings [10, 11]. It has been implicated that possible mechanisms involved in homocysteine-mediated vascular remodeling could be oxidative stress and decreased NO bioavailability [9, 12]; however, the mechanisms have not been well described.

Several studies indicated that hyperhomocysteinemia may be a possible component of the metabolic syndrome [13, 14]. Previous studies showed that hyperhomocysteinemia might induce insulin resistance [15]. Hyperhomocysteinemia may lead to altered cellular redox reactions [16, 17]. It may also affect NO bioproduction and NO bioavailability [18]. Oxidative stress and NO may be involved in the activation of matrix metalloproteinases (MMPs) [9, 19]. Hyperhomocysteinemia could cause increased deposition of collagen by the activation of MMPs [20, 21]. In addition, oxidative stress might be involved in disturbances of lipid and glucose metabolism in hyperhomocysteinemia [22].

Peroxisome proliferator activated receptor gamma (PPAR*γ*) is a nuclear receptor superfamily member, which may mitigate vascular complications. Homocysteine has been shown to antagonize PPAR*γ* and be inversely related to the expression of PPAR*γ* [23, 24], which could promote the synthesis of superoxide dismutase (SOD) and decrease oxidative stress [25, 26].

Because cardiovascular disease is a chronic disease, we used a rat model of long-term hyperhomocysteinemia to learn the long-term effect of hyperhomocysteinemia on metabolic parameters and vascular remodeling. Moreover, we studied oxidative stress and expression of PPAR*γ* in the coronary arteriole as a possible mechanism underlying vascular remodeling. In our study, the methionine supplementation was chosen to reflect the upper range that may be consumed additionally during overnutrition in human beings.

## 2. Materials and Methods

### 2.1. Animals and Treatments

All experiments were conducted in accordance with the National Institutes of Health Guide for the Care and Use of Laboratory Animals and approved by the Animal Care and Use Committee of Fudan University. Seven-week-old male Wistar rats from Shanghai Experimental Animal Center, Chinese Academy of Sciences, China, were used in the study. The rats were housed in polyethylene cages with a 12 h light-dark cycle and kept in a room at a constant temperature of 22 ± 3°C. Food and water were provided ad libitum. Body weight, food consumption, and water intake were monitored periodically. L-Methionine was purchased from Sigma-Aldrich (St. Louis, MO, USA).

After 7 days of acclimatization to the facility, the animals were randomized into two groups (*n* = 6 in each group): (1) the control-diet group was fed with standard rodent chow; (2) the Met-diet group was fed with the diet enriched in methionine (3%; wt/wt). All rats were killed after 48 weeks. Fasted blood glucose was measured upon the 48th week of diet following 6 h of fasting. Tail blood glucose concentrations were measured using a handheld glucometer (AccuCheck performa meter).

### 2.2. Serum, Spleen, and Heart Collection and Storage

After 48 weeks of diet, the animals were sacrificed under 10% chloral hydrate (350 mg/Kg, intraperitoneal injection) anesthesia. Abdominal aorta blood was collected, immediately cooled on ice, and centrifuged at 3000 rpm for 10 min at +4°C. Aliquots of serum layer were stored at −80°C until analysis. Then, the spleen, the heart, and carotid artery were removed. The spleen was weighed.

### 2.3. Measurement of Plasma Homocysteine

At the 48th week, the fasting animals were bled from the retroorbital plexus under aether anesthesia. Blood samples were obtained in chilled EDTA-containing microtubes and immediately centrifuged at 3000 rpm for 10 minutes at 4°C to limit the release of homocysteine (Hcy) from blood cells. Plasma was then stored at −80°C. Plasma Hcy concentration was measured by enzymatic cycling assay by Hitachi Model 7600 Series Automatic Analyzer (Hitachi High-Technologies Corporation, Japan).

### 2.4. Oral Glucose Tolerance Test (OGTT)

After 48 weeks of diet, OGTT was performed in rats after being fasted overnight. Glucose (3 g/Kg bodyweight glucose 500 g/l) solution was given orally to rats. Tail blood glucose concentration was determined at 0, 30, 50, 90, 120 minutes after administration.

### 2.5. Measurement of Serum Triglycerides, Total Cholesterol, High Density Lipoprotein, and Low Density Lipoprotein

The serum triglycerides, total cholesterol, high density lipoprotein (HDL), and low density lipoprotein (LDL) were measured using enzymatic assay kits (Nanjing Jiancheng Bioengineering Institute, Nanjing, China). All assays were performed according to the manufacturers' instructions.

### 2.6. Determination of Lipid Peroxidation and Superoxide Dismutase Activity

The degree of MDA in the serum was determined by measuring thiobarbituric acid reactive substances (TBARS) [27, 28]. A Malondialdehyde (MDA) Detection Kit (A003; Nanjing Jiancheng Bioengineering Institute, Nanjing, China) was used to determine the MDA level as a marker of lipid peroxidation. The Superoxide Dismutase Detection Kit (A001; Nanjing Jiancheng Bioengineering Institute, Nanjing, China) was used for SOD measurement. Both assays were conducted according to the manufacturer's instruction.

### 2.7. Measurement of Serum Nitric Oxide (NO)

The serum NO levels were detected by nitrate reductase method. The nitric oxide (NO) assay kit (A001; Nanjing Jiancheng Bioengineering Institute, Nanjing, China) was selected for NO measurement. The assay was conducted according to the manufacturer's instruction.

### 2.8. Histological Analysis

Coronal sections of ventricular myocardium and carotid artery were fixed in 10% neutral buffered formalin. The tissue sections (5 um) were stained with hematoxylin and eosin (H&E). Masson's trichrome stain was used for collagen and proteoglycans. Optical light microscopy was performed at 10x and 40x magnification. The coronary arteriolar wall-to-lumen ratio was analyzed in 50 to 200 um vessels. Percentages of collagen area of the carotid artery were calculated by dividing the area marked positive for collagen by the total tissue area.

### 2.9. Immunohistochemistry

The slices were dewaxed in xylene and hydrated in graded ethanol. Microwaves antigen retrieval was performed with citrate buffer for 10 min. Sections were washed in dH2O three times for 5 min each, incubated in 3% hydrogen peroxide for 10 min, washed in dH2O two times for 5 min each, and washed in wash buffer for 5 min. Then, each section was blocked with preferred blocking solution for 1 hour at room temperature. After blocking solution was removed, PPAR*γ* (Cell Signaling Technology, USA) diluted in antibody diluent was added as the primary antibody and incubated overnight at 4°C. Then antibody solution was removed and washed with wash buffer three times for 5 min each. Section was covered with horseradish peroxidase (HRP, Rabbit) as needed, incubated in a humidified chamber for 30 min at room temperature, and washed three times with wash buffer for 5 min each. DAB was applied to each section. The slides were washed with dH2O. Hematoxylin restaining, dehydration, transparent, sheet sealing, and microscopic examination were performed.

### 2.10. Statistical Analysis

Data were expressed as mean with standard errors (SEM) and statistical analysis was performed by SPSS 22.0 statistical software. All data were tested for normality prior to further analysis. Student's unpaired *t*-test was used to compare differences between groups. A *p* value of <0.05 was considered statistically significant.

## 3. Results

### 3.1. Plasma Homocysteine Levels

To confirm whether methionine administration induced an increase in plasma homocysteine, the plasma homocysteine level was measured. The plasma homocysteine level in the Met-diet group at the 48th week was significantly increased compared with the control-diet group at the same time (Table 1).

### 3.2. Physiological Variables after Methionine Loading

**Figure 1:**
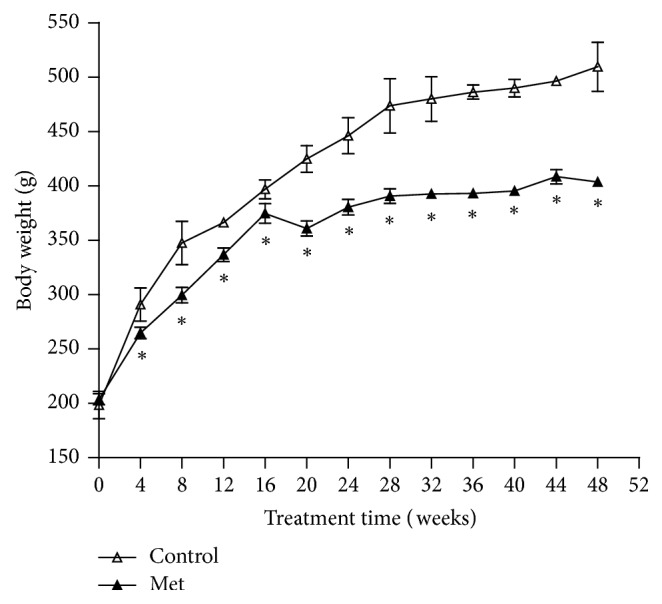
Effect of a control or methionine- (Met-) supplemented diet on change in body weight in Met-diet group (*n* = 6) and control-diet group (*n* = 6). Values are mean ± SEM. Mean values for the Met-diet group were significantly different from those of the control-diet group. ^*∗*^*p* < 0.05.

In the feeding period, the average water intake in Met-loaded rats was significantly increased compared with control animals (*p* < 0.05) (Table 1). There was no significant difference in the food consumption between the two groups (*p* > 0.05) (Table 1). The weight gain in Met-supplemented rats was slower than that in control rats (*p* < 0.05) (Figure 1). At the 48th week, the spleen weight/body weight in Met-supplemented rats was higher than that in control rats (*p* < 0.05) (Table 1).

### 3.3. Oral Glucose Tolerance Test

**Figure 2:**
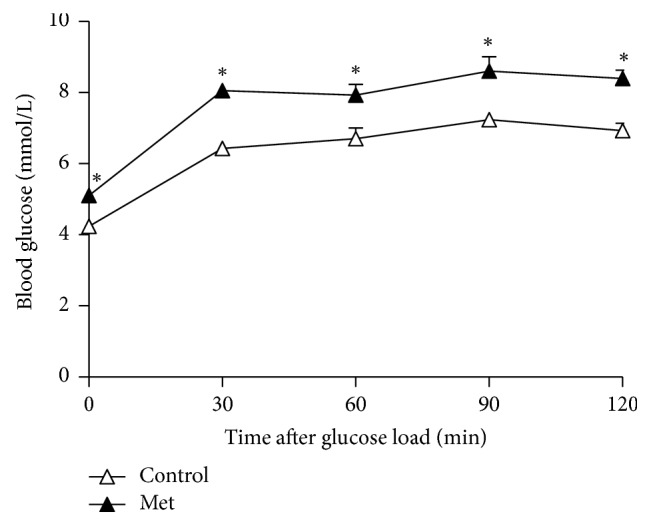
Glucose concentrations during the oral glucose tolerance test (OGTT) in the control-diet group (*n* = 6) and the Met-diet group (*n* = 6) at the 48th week. Values are mean ± SEM. ^*∗*^*p* < 0.005 versus the control-diet group.

We determined glucose tolerance by OGTT in each group at the 48th week. The glucose level rose to significantly higher concentrations in the Met-diet group compared with the control-diet group at 0 min, 30 min, 60 min, 90 min, and 120 min after glucose administration (*p* < 0.05) (Figure 2).

### 3.4. Metabolic Parameters

The results for metabolic measurements are shown in Table 1. At the 48th week, Met-supplemented rats showed an increase in the fasting blood glucose compared with control rats (*p* < 0.05). At the 48th week, the serum triglyceride level and the serum total cholesterol level in the Met-diet group were higher than those in the control-diet group (*p* < 0.05). However, there was no significant difference in the levels of the serum HDL and LDL between the two groups at the 48th week (*p* > 0.05).

### 3.5. Serum Malondialdehyde (MDA) and Superoxide Dismutase (SOD) Levels

Forty-eight weeks after methionine administration, the serum MDA levels were significantly increased and the serum SOD levels were significantly decreased in the Met-diet group compared with the control-diet group (*p* < 0.05) (Table 1).

### 3.6. Serum Nitrates (NO) Levels

The serum NO levels were significantly decreased in the control-diet group at the 48th week (*p* < 0.05) (Table 1).

### 3.7. Effect of Homocysteine on Carotid Arterial Remodeling

**Figure 3:**
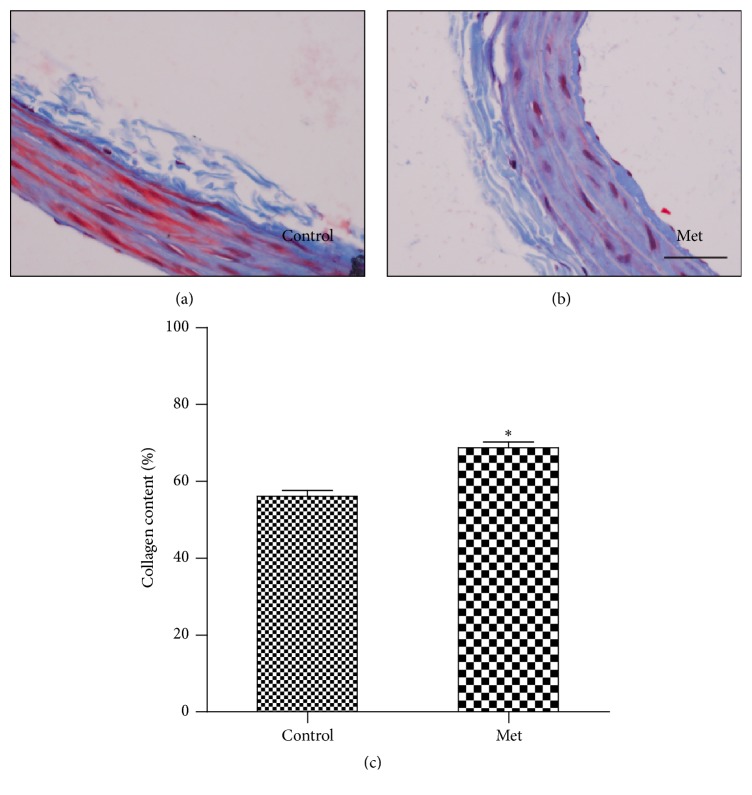
Histological analysis of carotid artery in the control-diet group and the Met-diet group. Tissue sections were labeled with trichrome (blue) for collagen. The staining showed hyperhomocysteinemia induced collagen accumulation in carotid artery in the Met-diet group (b) compared with representative vessels from the control-diet group (a). Collagen content was significantly increased in the Met-diet group (^*∗*^*p* < 0.05) (c). Original magnification was ×400 for (a)-(b) and the scale bar = 50 um. Values are mean ± SEM.

Carotid artery was stained with trichrome stain to detect the collagen deposition. Our result suggested that collagen content was significantly higher in the Met-diet group than the control-diet group, demonstrating arterial remodeling (Figure 3).

### 3.8. Effect of Homocysteine on Coronary Arteriolar Remodeling

**Figure 4:**
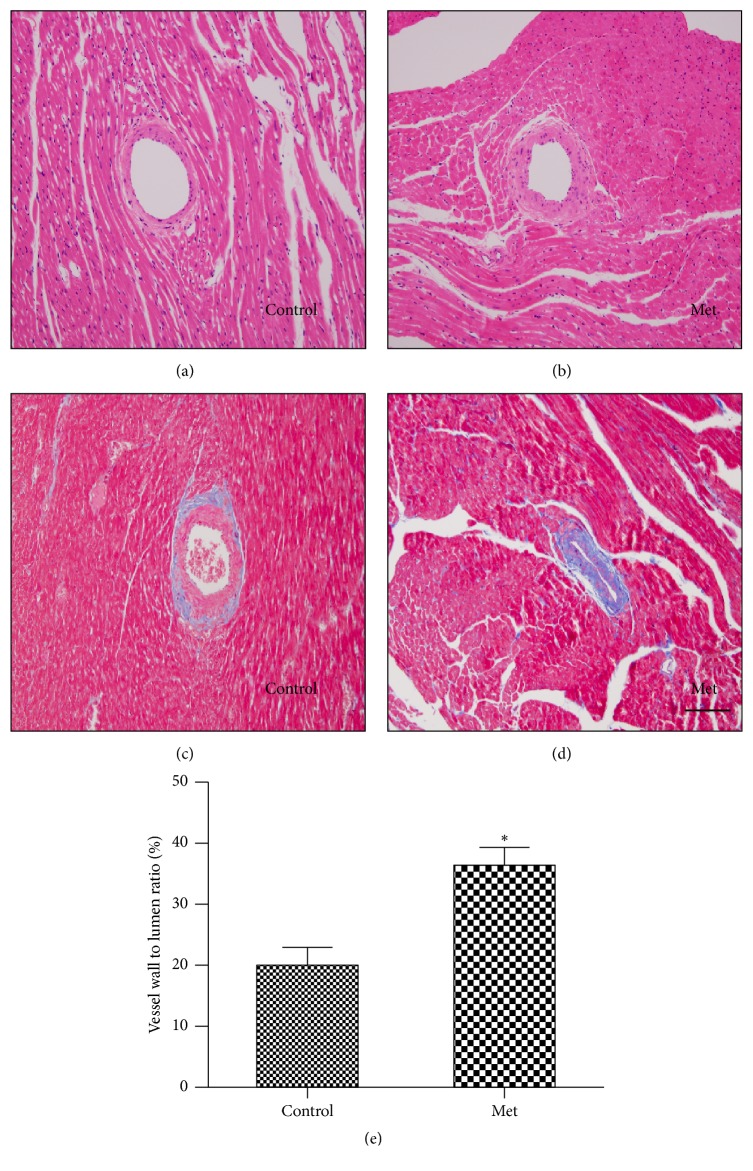
Representative images of stained heart sections. H&E stained sections from the control-diet group (a) and the Met-diet group (b) showed hyperhomocysteinemia induced coronary arteriolar wall thickening. Masson's trichrome staining showed hyperhomocysteinemia induced collagen accumulation in coronary arterioles in the Met-diet group (d) compared with vessels from the control-diet group (c). The wall-to-lumen ratios of coronary arterioles in the Met-diet group were increased compared with the control-diet group (^*∗*^*p* < 0.05) (e). Original magnification was ×100 for (a)–(d) and the scale bar = 100 um. Values are mean ± SEM.

To determine structural alteration by homocysteine, histological analysis was performed. Coronary arteriole was stained with H&E and trichrome (Figure 3). Coronary arteriolar wall thickness was increased in the Met-diet group compared with the control-diet group at the 48th week. The wall-to-lumen ratios of coronary arterioles in the Met-diet group were higher than those in the control-diet group (*p* < 0.05). The collagen deposition was increased in coronary arterioles of the Met-diet group compared with the control-diet group (Figure 4).

### 3.9. Effect of Homocysteine on PPAR*γ* Expression in the Coronary Arteriole

**Figure 5:**
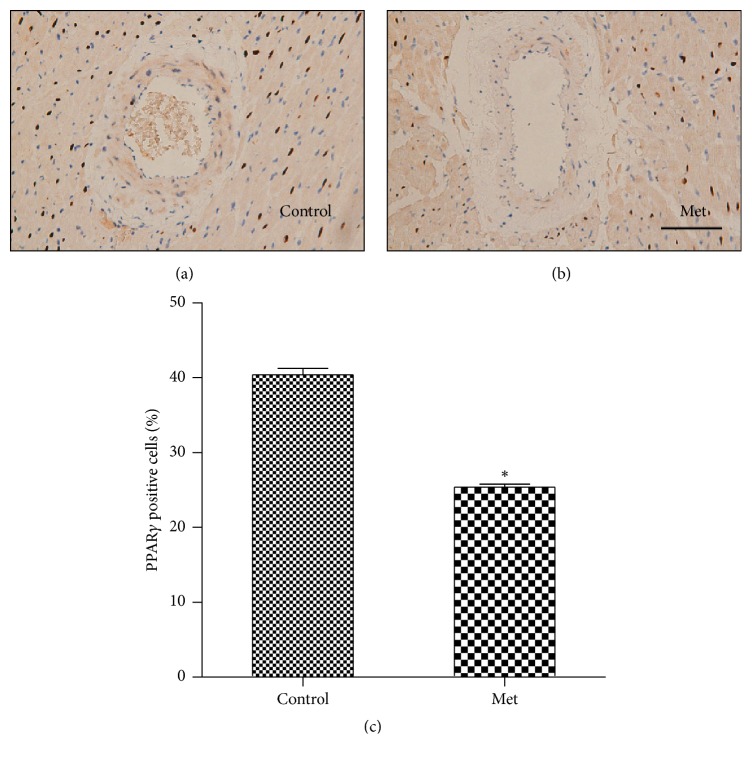
Representative immunohistochemical images of PPAR*γ* in the coronary arteriole in the control-diet group (a) and the Met-diet group (b). Positive staining of PPAR*γ* was found in the nuclei of the cells. The Met-diet group had significantly fewer PPAR*γ* positive cells (^*∗*^*p* < 0.05) (c). Original magnification was ×200 for (a)-(b) and the scale bar = 100 um. Values are mean ± SEM.

As shown in Figure 5, PPAR*γ* expression was found in the coronary arteriole of the control-diet group; PPAR*γ* expression decreased in the coronary arteriole of the Met-diet group (*p* < 0.05).

## 4. Discussion

In the current study, we found that long-term hyperhomocysteinemia could cause carotid arterial and coronary arteriolar remodeling due to collagen deposition in the vessels. We also found that PPAR*γ* expression decreased in the coronary arteriole. Moreover, our results showed that homocysteine could lead to disturbances of lipid and glucose metabolism, impaired oxidative stress, and endothelium dysfunction.

Results from our study showed disturbances of glucose metabolism after 48 weeks of administration of methionine diet. We speculated the disturbances of glucose metabolism may be associated with insulin resistance. Some studies suggested that hyperhomocysteinemia may induce insulin resistance [15, 29]. However, other studies showed that hyperinsulinemia can cause elevated plasma Hcy by impairing the activity of Hcy metabolizing enzymes [30, 31]. After 48 weeks of administration of methionine, the rats had significantly increased levels of plasma cholesterol and triglycerides. Previous studies also indicated that cholesterol and triglycerides were not significantly elevated in the plasma but significantly elevated in the livers of mice fed hyperhomocysteinemic diets for 10 to 20 weeks [32]. So hyperhomocysteinemia may cause ectopic accumulation of fat in liver at an early stage and disturbances of plasma lipid in the long run. The mechanisms between hyperhomocysteinemia and disturbances of lipid and glucose metabolism are not fully elucidated and may be associated with methyl group [33] and oxidative stress [22].

The present study demonstrated that plasma MDA levels were increased and SOD activity was decreased in the hyperhomocysteinemic rats. Our findings were consistent with previous studies [6, 34–36]. Oxidative radicals may be responsible for decreased production and bioavailability of endothelial-derived NO which may result in impaired endothelial-dependent vascular reactivity [37]. Hcy indirectly diminishes NO bioavailability by generating superoxide which rapidly reacts with NO causing the generation of peroxynitrite [38]. In this study, we demonstrated that hyperhomocysteinemia was associated with decreased NO levels, lower SOD activity, and increased MDA levels, suggesting that oxidative stress caused by Hcy may be related to the decreased NO bioavailability.

Oxidative stress may regulate the quantity and quality of extracellular matrix by activating matrix metalloproteinases (MMPs) [19]. Hcy can promote oxidative stress, thereby triggering the activation of MMPs [39]. NO may also have a role in the activation of MMPs [9]. The increased activity of MMPs can result in the degradation of extracellular matrix (ECM) components. Hence, Hcy may cause MMP-mediated degradation of ECM components and increased deposition of collagen, leading to the remodeling of the vessel wall [20, 21]. Some clinical studies indicated that hyperhomocysteinemia was a risk factor for increased carotid wall thickness [10, 11]. Our results indicated that Hcy caused the remodeling of the carotid artery after a long term of methionine-enriched diet, which may be related to the collagen deposition in the vessels, and so was the coronary arteriole.

In the present study, PPAR*γ* expression decreased in the coronary arteriole of the Met-diet group compared with the control group. It is known that PPAR*γ* induces SOD and decreases oxidative stress [25, 26]. On the other hand, previous study has suggested that homocysteine might induce vascular constrictive remodeling by antagonizing PPAR [40]. So we postulated that oxidative stress might activate MMPs leading to vascular remodeling by antagonizing PPAR*γ*. Our study may suggest a potential benefit of PPAR*γ* agonists on reversing the Hcy-mediated vascular remodeling in patients with hyperhomocysteinemia.

In our study, the spleens were enlarged after 48 weeks of feeding 3% methionine diets. The mechanism of which excessive methionine diet causes spleen hypertrophy is probably by changes in iron metabolism [41, 42].

There are some limitations in this study. First, we did not measure markers of oxidative stress (SOD, MDA) and MMPs in the carotid and coronary arteries. Second, the mechanisms of vascular remodeling remained unclear. Impaired oxidative stress, endothelium dysfunction, and decreased PPAR*γ* expression in the vessel wall may be involved, but further studies are needed to elucidate the underlying mechanisms.

## 5. Conclusion

In conclusion, the results of this study suggested that chronic hyperhomocysteinemia caused metabolic disturbances together with vascular remodeling and suggested that impaired oxidative stress, endothelium dysfunction, and decreased PPAR*γ* expression in the vessel wall could be underlying mechanisms.

## Figures and Tables

**Table 1:** Parameter values in Wistar rats after 48 weeks of treatment with control and methionine-supplemented diets (*n* = 6/group).

Parameters	Control-diet group	Met-diet group	*p*
Water intake (ml/d)	23.75 ± 1.69	31.29 ± 1.87^*∗*^	0.014
Food intake (g/d)	17.59 ± 0.65	16.10 ± 1.10	0.142
Spleen weight/body weight (mg/100 g)	111.51 ± 4.24	169.04 ± 7.53^*∗*^	0.001
Fasting blood glucose (mmol/L)	4.23 ± 0.08	4.93 ± 0.08^*∗*^	<0.001
Serum triglyceride (mmol/L)	0.29 ± 0.02	0.71 ± 0.03^*∗*^	<0.001
Serum total cholesterol (mmol/L)	1.37 ± 0.08	1.81 ± 0.04^*∗*^	<0.001
Serum high density lipoprotein (mmol/L)	0.73 ± 0.07	0.76 ± 0.05	0.753
Serum low density lipoprotein (mmol/L)	0.68 ± 0.07	0.77 ± 0.09	0.423
Serum NO (umol/L)	22.46 ± 1.05	8.22 ± 1.16^*∗*^	<0.001
Serum MDA (umol/L)	27.05 ± 4.51	137.18 ± 16.01^*∗*^	<0.001
Serum SOD (umol/L)	71.74 ± 1.64	20.31 ± 1.78^*∗*^	<0.001
Plasma homocysteine (umol/L)	3.00 ± 0.35	10.75 ± 0.80^*∗*^	<0.001

Met, methionine. Data are presented as mean ± SEM. ^*∗*^Significantly different from corresponding values in the control group.
